# A review of advances in the understanding of lupus nephritis pathogenesis as a basis for emerging therapies

**DOI:** 10.12688/f1000research.22438.1

**Published:** 2020-08-04

**Authors:** Susan Yung, Desmond YH Yap, Tak Mao Chan

**Affiliations:** 1 Department of Medicine, The University of Hong Kong, Pokfulam, Hong Kong

**Keywords:** lupus nephritis, lupus nephritis pathogenesis, inflammation, B cell depletion, emerging therapies

## Abstract

Lupus nephritis is an important cause of both acute kidney injury and chronic kidney disease that can result in end-stage renal disease. Its pathogenic mechanisms are characterized by aberrant activation of both innate and adaptive immune responses, dysregulation of inflammatory signaling pathways, and increased cytokine production. Treatment of lupus nephritis remains a challenging issue in the management of systemic lupus erythematosus since the clinical presentation, response to treatment, and prognosis all vary considerably between patients and are influenced by ethnicity, gender, the degree of chronic kidney damage, pharmacogenomics, and non-immunological modulating factors. Elucidation of the various immunopathogenic pathways in lupus nephritis has resulted in the development of novel therapies, including biologics that target specific antigens on B lymphocytes to achieve B cell depletion, agents that modulate B cell proliferation and development, drugs that block co-stimulatory pathways, drugs that target T lymphocytes primarily, and therapies that target complement activation, signaling pathways, pro-inflammatory cytokines, and neutrophil extracellular traps. This review will discuss recent advances in the understanding of disease pathogenesis in lupus nephritis in the context of potential emerging therapies.

## Introduction

**Figure 1.**
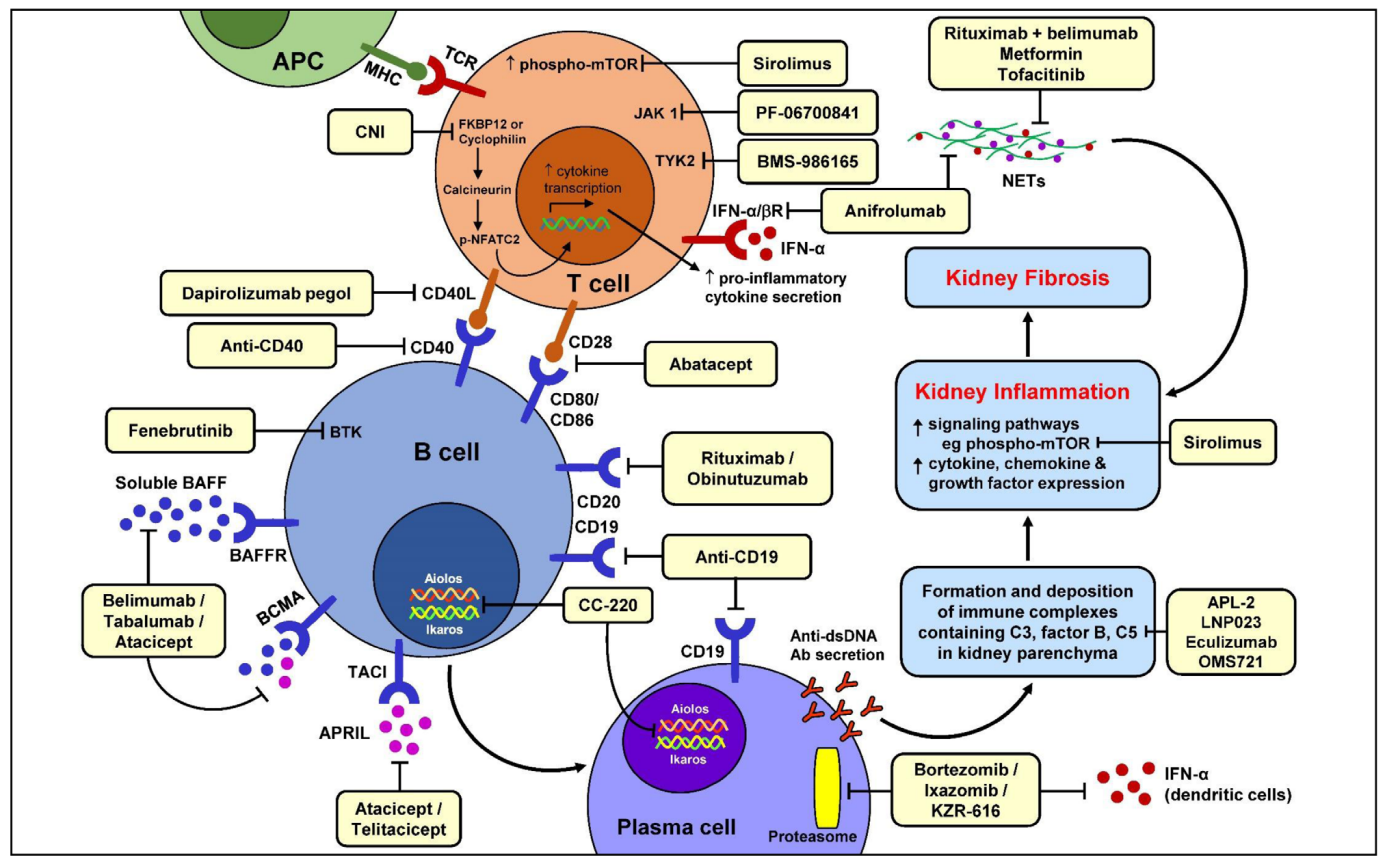
Schematic diagram showing the immunopathogenic pathways leading to kidney damage in lupus nephritis and emerging novel therapies targeting specific pathways. Ab, antibody; APC, antigen presenting cell; APRIL, a proliferating-inducing ligand; BAFF, B cell activating factor; BAFFR, B cell activating factor receptor; BCMA, B cell maturation antigen; BTK, Bruton’s tyrosine kinase; C3, complement 3; C5, complement 5; CD, cluster of differentiation; CNI, calcineurin inhibitor; ds, double-stranded; FKBP12, FK506-binding protein; IFN, interferon; IFNα/βR, interferon-α/β receptor; JAK, Janus kinase; MHC, major histocompatibility complex; mTOR, mammalian or mechanistic target of rapamycin; NETs, neutrophil extracellular traps; NFATC2, nuclear factor of activated T cells 2; TACI, transmembrane activator and calcium modulator and cyclophilin ligand interactor; TCR, T cell receptor; TYK2, tyrosine kinase-2.

Kidney involvement, termed lupus nephritis (LN), is one of the most severe forms of organ involvement in systemic lupus erythematosus (SLE) as reflected by the associated morbidities, its detrimental effects on patient and kidney survival, and the quantity of immunosuppressive agents required for treatment ^1, 2^. The resultant chronic kidney damage and the exposure to toxic medications are also significant contributors to reduced survival of patients even after successful treatment of active nephritis. The pathogenesis of LN encompasses a loss of self-tolerance, aberrant activation of both innate and adaptive immune responses, autoantibody production, and immune-mediated kidney injury. LN is characterized by episodic flares separated by variable durations of disease quiescence. Even when immunosuppressive treatments are successful in inducing remission, the cumulative kidney damage resulting from repeated nephritic flares, as evident from glomerulosclerosis, fibrous crescents, tubular atrophy, and interstitial fibrosis in the kidney biopsy, portends progressive chronic kidney disease eventually culminating in end-stage renal failure ^3^. The goals of clinical management therefore include early detection and prompt and effective treatment of acute nephritic flares as well as the prevention of relapses. The efficacy and tolerability of current immunosuppressive treatments for LN vary between patients and are influenced by multiple factors including race and renal reserve. In addition, corticosteroids remain a mainstay of treatment but are associated with considerable short-term and long-term toxicities related to non-specific anti-inflammatory and immunosuppressive actions. It is against this background that the search for novel therapies has continued with the aim of adding new treatments with increased levels of safety, often related to increased specificity in intercepting crucial pathogenic pathway(s), to the therapeutic armamentarium. This review presents a brief overview of recent knowledge in the pathogenesis of LN in the context of potential emerging therapies ( Figure 1) ( Table 1).

**Table 1.** Examples of novel therapies for systemic lupus erythematosus targeting specific cells or pathogenic pathways.

Target	Drug	Study outcomes and ongoing trials
**B cells (CD20)**	Rituximab (chimeric anti-CD20 mAb) Obinutuzumab (humanized type 2 anti-CD20 mAb)	• Data from the LUNAR trial showed that rituximab, when added to standard induction treatment, did not show renal benefits in active LN patients • May have a role in refractory or frequently relapsing disease, high-risk patient groups (e.g. Afro-Americans), or corticosteroid minimization • A phase II trial (NOBILITY, NCT02550652) showed that obinutuzumab, when added to background immunosuppression comprising mycophenolate and corticosteroids, increased the rate of achieving primary and secondary efficacy endpoints, possibly related to a rapid and complete depletion of peripheral, memory, and naïve B cell subsets and plasmablasts
**B cells (CD19)**	XmAb5871 (humanized anti- CD19 mAb)	• A phase II clinical trial (NCT02725515) showed that XmAb5871 treatment in non-organ-threatening SLE patients did not meet the primary endpoint (no LOI) but achieved the secondary endpoints (time-to-LOI and safety)
**B cells (BAFF/** **APRIL)**	Belimumab (humanized anti- BAFF mAb) Tabalumab (humanized mAb against soluble and membrane-bound BAFF) Atacicept (fusion protein between TACI and Fc portion of IgG) Telitacicept (novel recombinant TACI-Fc fusion protein)	• Data from BLISS-52 and -76 trials showed efficacy in SLE patients, but patients with moderate-to-severe LN or neuropsychiatric complications were excluded • Results from BLISS-LN (NCT01639339) showed that patients with active LN treated with belimumab plus standard induction treatment had superior renal response compared with placebo • RCT data from ILLUMINATE-1 and -2 showed that tabalumab did not meet primary efficacy endpoint SRI-5 at 52 weeks in patients with active SLE without nephritis; pooled data from these two RCTs also did not show any renal benefits • A phase II/III clinical trial in patients with active LN (APRIL-LN; NCT00573157) was terminated early owing to severe infective complications associated with hypogammaglobulinemia • A phase IIb study (NCT02885610) showed that telitacicept in combination with standard therapy met its primary endpoints in active SLE patients without nephritis
**B cells** **(Proteasome)**	Bortezomib Ixazomib KZR-616	• Preliminary data showed efficacy in patients with refractory LN but was associated with high rates of treatment discontinuation because of poor drug tolerability • A phase II study (NCT02102594) is currently underway to investigate its use in SLE patients • Ixazomib shows fewer neurological side effects compared to bortezomib, and a phase II study (NCT02176486) is currently underway to investigate its use in LN patients • Phase I data from MISSION (NCT03393013) suggest that KZR-616 is well tolerated in SLE patients with or without nephritis; a phase II study has been initiated to investigate its use in LN patients
**B cells (Aiolos** **and Ikaros)**	Iberdomide (CC-220) (a cereblon modulator targeting Ikaros and Aiolos)	• A phase IIa study (NCT02185040) showed that iberdomide (CC-220) reduced various B cell subsets and improved arthritis and CLASI activity scores in SLE patients
**T cells** **(Calcineurin)**	Tacrolimus Voclosporin	• Results from clinical trials in LN patients showed that dual or triple immunosuppressive regimens that included tacrolimus were associated with comparable or higher short-term response rate and often more rapid reduction of proteinuria compared to treatment regimens that did not include a CNI • Improved pharmacokinetic profile compared to tacrolimus • A phase II study (AURA-LV; NCT02141672) in active LN patients showed that voclosporin (high or low dose) when combined with corticosteroids and MMF showed superior renal response rates compared with corticosteroids and MMF treatment • A phase III study (AURORA; NCT03021499) reported that voclosporin (23 mg bid) in combination with corticosteroids (rapid tapering) and MMF in active LN patients resulted in better renal response rates and proteinuria suppression compared with control treatment comprising corticosteroids and MMF
**Co-stimulatory** **pathways (CTLA4)**	Abatacept	• Phase II data showed that induction therapy for active proliferative LN with abatacept combined with corticosteroids and MMF improved proteinuria and serological parameters but did not meet primary endpoint (time to complete renal response) for superiority • The phase III ALLURE trial reported that the addition of abatacept to standard of care in active LN patients did not increase renal response rate, but those who received abatacept appeared to show more rapid and more sustained proteinuria reduction
**Co-stimulatory** **pathways (CD40)**	BI655064 (humanized anti- CD40 mAb) CFZ533 (humanized anti-CD40 mAb)	• A phase II clinical trial (NCT02770170) is currently underway to investigate the efficacy and safety of BI655064 in combination with standard of care therapy in active LN patients • A phase II study (NCT03610516) is currently underway to investigate its use in LN patients who show persistent significant proteinuria after standard induction treatments
**Co-stimulatory** **pathways (CD40L)**	Dapirolizumab pegol (pegylated Fab anti-CD40L)	• A recent phase II trial (NCT02804763) showed numerically higher response rates and greater improvement in several clinical outcome measures compared to placebo but did not meet the primary endpoint (BICLA response rates at 24 weeks)
**mTOR**	Sirolimus	• A recent single-arm phase I/II study (NCT00779194) showed that sirolimus treatment in active SLE patients without nephritis or life-threatening lupus complications had decreased disease activity scores and T cell profile indicating a less pro-inflammatory phenotype • Data from our group show that sirolimus may have a role in LN management, especially in LN patients who do not tolerate other treatments, and may be considered especially in patients with a history of malignancy
**Type I IFNR**	Anifrolumab (humanized mAb to type I IFNR)	• Data from phase III TULIP-1 and -2 trials showed clinical benefits of anifrolumab treatment in moderate-to-severe SLE patients with active disease, especially in those with high type I IFN signatures • Decreased NET formation
**IL-23**	Guselkumab (humanized anti- IL-23 mAb)	• A forthcoming phase II study will investigate its application in LN
**IL-17**	Secukinumab (humanized anti- IL-17 mAb)	• A phase II clinical study (NCT04181762) is underway to investigate its role in patients with refractory LN
**IL-2**	Low-dose IL-2	• One recent RCT (NCT02465580) showed that low-dose IL-2 treatment in active SLE patients did not meet its primary endpoint of SRI-4 at 12 weeks but achieved higher complete remission rates than placebo in patients with LN and was well tolerated
**JAK1**	Baricitinib (JAK1/2 inhibitor) PF-06700841 t(selective JAK1 inhibitor)	• A phase II RCT (NCT02708095) demonstrated efficacy in alleviating arthritis in active SLE patients • A phase IIb study (PF-06700841) will investigate a selective JAK1 inhibitor in moderate-to-severe active SLE patients without renal or cerebral involvement who show inadequate response to standard therapies • A phase II clinical trial on solcitinib (a selective JAK1 inhibitor) (NCT01777256) was discontinued owing to severe drug reaction with eosinophilia and systemic symptoms (DRESS) syndrome and hepatic function abnormalities
**TYK2**	BMS-986165 (an oral selective TYK2 inhibitor)	• BMS-986165 blocks signaling pathways of multiple cytokines (IL-12, IL-23, and type I IFN) • A phase II RCT (PAISLEY; NCT03252587) is underway to investigate its efficacy and safety in active SLE patients with joint and/or skin involvement
**BTK**	Fenebrutinib (BTK inhibitor)	• Results from a phase II RCT (NCT02908100) showed that fenebrutinib treatment in patients with moderate or severe active SLE was associated with a significant reduction in CD19+ B cells, anti-dsDNA levels, and BTK-dependent signature in plasmablasts, but the primary clinical efficacy endpoint (SRI-4) was not met
**Complement**	APL-2 (synthetic peptide that binds C3 and C3b) Eculizumab (anti-C5b monoclonal antibody) LNP023 (factor B inhibitor) OMS721 (MASP-2 inhibitor)	• Data from a phase II clinical trial showed that APL-2 was well tolerated and treatment was associated with a reduction in urine protein excretion • Phase I study showed that eculizumab was well tolerated in SLE patients • Emerging data suggest efficacy in selected LN patients such as those with TMA or refractory nephritis • Phase II trial in patients with paroxysmal nocturnal hemoglobinuria and IgA nephropathy but has not been investigated in LN • Phase II study showed that OMS721 treatment of LN patients was associated with significant reduction in proteinuria and feasibility of steroid dose tapering
**NETs**	B cell depletion Tofacitinib (JAK/STAT inhibitor) Metformin	• SynBioSe study (NCT02284984) showed that combination treatment with rituximab and belimumab reduced NET formation in SLE patients • Phase I study will investigate the safety and tolerability of tofacitinib in SLE patients • A study in Chinese patients (ChiCTR-TRC-12002419) showed that metformin added to conventional treatment for 12 months in SLE patients with mild-to-moderate disease decreased SLEDAI score by over 50% • Metformin reduced NET formation in neutrophils stimulated with PMA and decreased IFN-α secretion in plasmacytoid dendritic cells

APRIL, a proliferating-inducing ligand; BAFF, B cell activating factor; BICLA, British Isles Lupus Assessment Group-based Combined Lupus Assessment; bid, *bis in die* (twice a day); BTK, Bruton’s tyrosine kinase; C3, complement 3; C5, complement 5; CLASI, cutaneous lupus erythematosus disease area and severity index; CNI, calcineurin inhibitor; CTLA4, cytotoxic T-lymphocyte-associated-antigen 4; DRESS, drug rash with eosinophilia and systemic symptoms; ds, double-stranded; IFN, interferon; IFNR, interferon receptor; Ig, immunoglobulin; IL, interleukin; JAK, Janus kinase; LN, lupus nephritis; LOI, loss of improvement; mAb, monoclonal antibody; MASP-2, mannan-binding lectin serine protease 2; MMF, mycophenolate mofetil; mTOR, mammalian or mechanistic target of rapamycin; NET, neutrophil extracellular trap; PMA, phorbol 12-myristate 13-acetate; RCT, randomized controlled trial; SLE, systemic lupus erythematosus; SLEDAI, systemic lupus erythematosus disease activity index; SRI, systemic lupus erythematosus responder index; STAT, signal transducer and activator of transcription; TACI, transmembrane activator and calcium modulator and cyclophilin ligand interactor; TMA, thrombotic microangiopathy; TYK2, tyrosine kinase 2.

## B lymphocytes in the pathogenesis of lupus nephritis

The etiology of LN is complex and multi-factorial and involves the interplay between genetic predisposition and environmental and hormonal factors ^4– 7^. LN is characterized by the production of autoantibodies against a broad diversity of autoantigens, in particular against chromatin material such as double-stranded (ds) DNA and nucleosomes resulting from defective clearance of apoptotic material ^8^, and necrotic cells that release cellular components which may form neoantigens. The release of DNA/protein and RNA/protein from dying cells activates dendritic cells, monocytes, and macrophages through Toll-like receptor (TLRs), resulting in the secretion of pro-inflammatory mediators, such as interferon (IFN)-α, tumor necrosis factor (TNF)-α, and interleukin (IL)-6, which activate effector T cells and B cells. Aberrant T cell activation and the prolonged survival and maturation process of B cells result in increased numbers of autoreactive B cells, memory B cells, and plasma cells. Abnormalities in B cell biology in SLE include early entrance of immature, transitional, and naïve B cells to mature B cells, attributed in part through increased expression of B-cell-activating factor (BAFF), a cytokine that promotes B cell survival from late transitional stage to mature and memory B cells. FcγRIIIB expression is reduced in SLE patients compared to healthy subjects, resulting in the persistence of autoreactive B cells. Also, memory B cells in SLE patients show reduced FcγRIIB expression and a lower threshold for reactivation ^9^. B cells derived from SLE patients exhibit increased somatic hypermutation and class-switch recombination, resulting in enhanced pathogenicity of plasma cells ^10– 12^. Autoantibodies produced by plasma cells in LN patients are usually of the immunoglobulin (Ig) G subclass, and the isotype can be related to the nature of the respective antigen. For example, protein and polysaccharide antigens have been shown to induce IgG1 and IgG2, respectively ^13^. Also, pro-inflammatory cytokines such as IL-4 and IL-21 can induce isotype switching ^13, 14^. In this regard, IgG1 and IgG3 bind FcγR more efficiently to trigger complement activation, downstream inflammatory processes, immune complex deposition, and tissue injury.

## Emerging therapies targeting B lymphocytes

As described previously, B cells are promising therapeutic targets of LN since they are central to pathogenesis ^15, 16^. The pathogenic role of B cells is not just limited to autoantibody production but extends to antigen presentation, T cell activation and polarization, modulation of dendritic cell maturation, and cytokine secretion ^15, 16^. The survival and maturation of B cells at different stages of development depend on the delivery of survival and trophic signals through cell surface ligands such as BAFF, CD19, and CD20. These cell surface antigens may thus serve as therapeutic targets for B cell depletion in the treatment of SLE and LN.

### Rituximab

CD20 is a specific B cell surface antigen that promotes differentiation and activation. It is expressed on immature, mature, and activated B cells and is absent on hematopoietic stem cells, pro-B cells, and plasma cells ^17, 18^. Rituximab is a type I chimeric IgG1 mouse/human monoclonal antibody directed against CD20, and it depletes B cells, thus diminishing their differentiation into plasma cells, and therefore decreases autoantibody production ^19^. Rituximab was originally approved for the treatment of relapsed or refractory, low-grade or follicular, CD20 ^+^ B cell non-Hodgkin lymphoma, and its use has been extended to various autoimmune diseases such as rheumatoid arthritis, ANCA-associated vasculitis, and primary immune thrombocytopenic purpura ^20^. Crosslinking of CD20 molecules by rituximab induces the redistribution of CD20 into membrane microdomains known as lipid rafts and the formation of antigen–antibody complexes, leading to CD20 ^+^ B cell apoptosis through three mechanisms, namely complement-dependent cytotoxicity, antibody-dependent cell-mediated cytotoxicity, and, to a lesser extent, direct signaling through CD20. Rituximab eliminates peripheral B cells without suppressing their regeneration from B cell precursors, and, by sparing plasma cells, has no immediate effect on Ig level. In preclinical studies, treatment of New Zealand black and white, first generation (NZB/W F1) mice with anti-CD20 antibodies before the onset of proteinuria delayed disease onset, whereas treatment after onset of nephritis reduced disease progression, suggesting that B cells are critical to both the initiation and the maintenance of disease ^21^. There is also a subset-associated hierarchy of B cell sensitivity to depletion by rituximab, with peripheral blood B cells being the most sensitive, and B cells in the spleen, lymph node, or bone marrow being less sensitive, which could be related to relatively low tissue penetration by the antibody. Despite its primary action on B cells, the B cell depletion that resulted from treatment with rituximab was associated with a reduction in T cell memory and activation. Some NZB/W F1 mice appeared to be resistant to the B cell-depleting action of rituximab, which was attributed to a rapid clearance of circulating anti-CD20 antibodies and/or increased BAFF secretion ^21^.

Data from the LUNAR trial showed that rituximab, when added to standard induction immunosuppressive regimens, did not increase the rate of renal response, despite leading to more pronounced improvements in serological parameters and proteinuria ^22^. Proposed explanations for this apparent paradox include the efficacy of background immunosuppression, suboptimal depletion of tissue-infiltrating B cells and plasma cells, increased BAFF secretion, and inadequate sensitivity of clinical efficacy endpoints. Lack of treatment efficacy could also be related to monoclonal antibody metabolism, reduced antibody binding due to FcγRIII polymorphism, complement depletion, increased expression of complement inhibitory molecules such as CD55 and CD59, abnormal lipid raft composition, or limited access to the parenchyma of solid organs ^23, 24^. In this regard, B cells are present in the tubulo-interstitium and contribute to local inflammation and kidney injury ^25^. It is currently unknown to what degree rituximab can directly deplete B cells in the kidney. Another mechanism for rituximab resistance may involve the upregulation of CD46, a key surface protein that inhibits complement activation. Serum CD46 level, possibly a product of proteolytic cleavage from apoptotic cell membranes, is increased in SLE patients with active disease compared to patients with inactive disease ^26, 27^. Whether combination therapy targeting both CD46 and CD20 may increase efficacy remains to be investigated. Despite the negative results in pivotal trials, rituximab is commonly used in the management of SLE patients, including patients with LN, in view of the favorable real-world clinical experience in severe renal or extra-renal manifestations and its relatively low toxicity, especially in patients who have an inadequate response to or who cannot tolerate standard therapies. Results from post-hoc analysis of the LUNAR trial also suggested that it may be particularly useful in high-risk patient groups such as Afro-Americans ^28– 32^.

### Obinutuzumab

Obinutuzumab is a glycoengineered, type II, humanized anti-CD20 antibody developed to treat lymphoproliferative disorders and to alleviate several mechanisms which could lead to treatment resistance. Obinutuzumab has no fucosylated sugars on the Fc portion and demonstrates enhanced binding affinity to the FcγRIII receptor on immune effector cells ^24^. Obinutuzumab is more effective than rituximab in inducing direct B cell death and antibody-dependent cellular cytotoxicity and phagocytosis. Unlike rituximab, it does not relocalize CD20 to lipid rafts and its effect on B cell depletion is not influenced by complement protein exhaustion or complement-inhibiting factors ^24^. Pre-clinical data showed the superior efficacy of obinutuzumab in B cell depletion compared with rituximab ^24^. Recent results from the phase II NOBILITY study (NCT02550652) showed that obinutuzumab, when added to background immunosuppression comprising corticosteroids and mycophenolate, increased the rates of achieving primary and secondary efficacy endpoints, possibly related to a rapid and complete depletion of peripheral, memory, and naïve B cell subsets and plasmablasts ^33, 34^.

### Belimumab and agents that target the BAFF/APRIL pathway

BAFF and a proliferation-inducing ligand (APRIL) are transmembrane proteins of the TNF family synthesized by myeloid cells. They can be proteolytically cleaved from the cell and exist in soluble form. BAFF and APRIL bind to receptors such as transmembrane activator and calcium modulator and cyclophilin ligand interactor (TACI) and B-cell maturation antigen (BCMA). BAFF and APRIL are required for B cell survival from their initial development to terminal differentiation. BAFF promotes the survival and maturation of transitional B cells into mature B cells and supports B cell proliferation, plasma cell survival, and class-switch recombination. APRIL also promotes cell survival and class-switch recombination and plays an important role in T cell-independent responses ^13^. Inhibition of B cell survival factors represents an alternative approach to inhibit B cell and humoral immune responses. Belimumab is a human IgG1λ antibody that blocks the bioactivity of soluble BAFF, thus inhibiting B cell survival and differentiation. Results from two randomized controlled trials (RCTs), BLISS-52 and BLISS-76, demonstrated the efficacy of belimumab in active SLE patients, but patients with moderate or severe LN or neuropsychiatric complications were excluded from these trials ^35, 36^. Post-hoc analysis of the pooled data from these trials suggested an efficacy of belimumab treatment on renal manifestations and a decreased incidence of renal flare in patients who received belimumab ^37^. Whether the latter could be explained by sustained inhibition of memory B cells and plasma cells remains speculative ^38^. A phase III clinical trial which investigated the efficacy of belimumab in LN (BLISS-LN; NCT01639339) was completed recently, and the results showed that a higher number of patients met its primary and secondary endpoints when treated with belimumab plus standard induction therapy compared to placebo and standard induction therapy ^39^.

While data on belimumab in SLE and LN appear to be encouraging, other anti-BAFF therapies have failed to demonstrate efficacy in clinical trials. Results from the ILLUMINATE-1 and -2 trials showed that treatment with tabalumab, a monoclonal antibody against both soluble and membrane-bound BAFF, did not meet the primary efficacy endpoint of SLE responder index (SRI)-5 in patients with active SLE but no renal involvement ^40, 41^, and pooled data from these two RCTs did not suggest beneficial effects of tabalumab on the renal outcomes ^42^. Atacicept is a human recombinant fusion protein of TACI and the Fc portion of IgG1. The APRIL-LN trial (NCT00573157), which aimed to investigate the role of atacicept in the treatment of active LN, was terminated early because of severe infective complications associated with low serum Ig level in the first few treated patients ^43^. Telitacicept, also known as RC18, is a novel recombinant TACI-Fc fusion protein that targets and inhibits BLyS (aka BAFF) and APRIL and suppresses the development and survival of plasma cells and mature B cells. A recent phase IIb study (NCT02885610) demonstrated that telitacicept in combination with standard therapy met its primary endpoint in active SLE patients without nephritis ^44^. Sequential rituximab-belimumab treatment in patients with active LN is a therapeutic approach currently being investigated (CALIBRATE; NCT02260934), aiming to ensure adequate B cell suppression by first depletion and then preventing their repopulation using belimumab ^45^. Interim data from the CALIBRATE study showed that despite B cell depletion and delayed circulating B cell reconstitution, sequential rituximab-belimumab treatment did not improve clinical outcome ^45^. Results from the SynBioSe study (NCT02284984) demonstrated that combination treatment with rituximab and belimumab in SLE patients with severe and refractory disease improved clinical outcome, and this was associated with decreased production of neutrophil extracellular traps (NETs) ^46^. A phase II clinical trial (SynBioSe-2; NCT03747159) will investigate the efficacy of belimumab followed by rituximab in LN patients. A phase III clinical trial (BLISS-BELIEVE; NCT03312907) investigating the efficacy and safety of belimumab administered in combination with rituximab in patients with active SLE is ongoing ^47^. Another ongoing study (phase II; NCT04058028) is investigating the use of BAFF/inducible T-cell co-stimulator ligand (ICOSL) bispecific antibody in active SLE patients without recent cerebral or renal involvement.

### Other therapies targeting B cells or plasma cells

B cell depletion can also be achieved by monoclonal antibodies binding to other B cell surface molecules such as CD19 (XmAb5871). In a recent phase II clinical trial (NCT02725515), XmAb5871 treatment of non-organ-threatening SLE patients did not meet the primary endpoint of “no loss of improvement (LOI)” but achieved the secondary endpoint of “time-to-LOI and safety” ^48^. Emerging evidence suggests that proteasome inhibitors such as bortezomib and ixazomib (an oral proteasome inhibitor with fewer neurological complications, such as a lower risk of peripheral neuropathy) can target plasma cells through binding to 26S proteasome and inhibiting its chymotrypsin-like activity and inactivating the nuclear factor kappa-light chain enhancer of activated B cell process. Preliminary clinical experience showed the efficacy of bortezomib in active LN patients who were refractory to conventional induction therapies ^49, 50^, although the high rates of treatment discontinuation due to poor drug tolerability remains a concern. Phase II clinical studies are currently underway to investigate the use of bortezomib in SLE patients (NCT02102594) and ixazomib in LN patients (NCT02176486). The MISSION study (NCT03393013) is a phase Ib/II trial that investigates the safety and tolerability of KZR-616, a specific immunoproteasome inhibitor, in patients with active SLE with or without LN (phase Ib) or patients with active proliferative LN (phase II). Results from the phase Ib study showed that KZR-616 at a dose of 45 mg SC was safe and well tolerated ^51^, and the phase II clinical trial is still ongoing.

Aiolos and Ikaros are members of the Ikaros family of hemopoietic-specific zinc-finger transcription factors that play key roles in the regulation of lymphocyte development and differentiation ^52, 53^. Ikaros is expressed in pluripotent hemopoietic stem cells and regulates both lymphoid and myeloid cell development. Through its ability to regulate the development of plasmacytoid dendritic cells, Ikaros can also regulate key inflammatory pathways such as signal transducer and activator of transcription (STAT) 4 and IFN-α pathways ^54^. Aiolos contributes to B cell development and facilitates the generation of long-lived high-affinity bone marrow plasma cells. Aiolos is weakly expressed in pro-B cells and is increased in pre-B cells and mature peripheral B cells ^55^. Studies have shown that Aiolos expression is increased in peripheral blood mononuclear cells (PBMCs) from SLE patients compared to healthy subjects ^56, 57^. CC-220 (iberdomide) is a modulator of the cullin ring ligase 4-cereblon (CRL4 ^CRBN^) E3 ubiquitin ligase complex, which induces ubiquitination of the CRBN substrates Aiolos and Ikaros, resulting in their proteasomal degradation. *In vitro* studies demonstrated that the addition of iberdomide to B cells or PBMCs from SLE patients or healthy subjects, respectively, reduced Aiolos and Ikaros expression and inhibited BAFF-induced B cell differentiation and the production of anti-dsDNA antibodies and anti-phospholipid antibodies ^56, 57^. A phase IIa clinical study (NCT02185040), which investigated the tolerability and effect of iberdomide on skin, joint, and serological manifestations in SLE patients, reported that iberdomide significantly reduced B cell subset populations and plasmacytoid dendritic cells and improved arthritis and cutaneous lupus erythematosus disease area and severity index (CLASI) activity score, an assessment score for cutaneous involvement ^58, 59^. A phase IIb clinical study (NTC03161483) to investigate the efficacy and safety of iberdomide in a larger cohort of patients with active SLE is ongoing.

## Emerging therapies targeting T lymphocytes

T lymphocytes in lupus patients exhibit a lower activation threshold compared with T cells in healthy subjects ^60^. T cell receptor (TCR)-mediated activation results in increased calcium influx and activation of calcineurin, a calcium–calmodulin-dependent serine-threonine phosphatase. Calcineurin activation leads to nuclear factor of activated T cells (NFAT) dephosphorylation, nuclear translocation of NFAT, and downstream induction of IFN-γ, TNF-α, and IL-2, IL-4, IL-6, and IL-17 ^61^. Calcineurin inhibitors (CNIs) such as cyclosporine A (CsA), tacrolimus (TAC), or voclosporin inhibit the T-cell-mediated immune response by binding to cytosolic immunophilins such as cyclophilins or FK506-binding protein (FKBP12), then inhibit calcineurin activity and NFAT dephosphorylation ^61^. Triple immunosuppression that includes corticosteroids, CNIs, and mycophenolate is the standard-of-care immunosuppressive regimen to prevent kidney transplant rejection ^62^. The same approach has been used in animal studies and clinical practice in the management of SLE. Treatment of MRL/lpr mice with prednisolone, TAC, and mycophenolate showed increased efficacy compared with monotherapy, and transcriptomic analysis demonstrated synergistic actions of the different drugs targeting different disease-relevant pathogenic pathways ^63^. In addition to its action on T cells, CNIs modulate the podocyte cytoskeleton through synaptopodin-regulated Ras homolog family member A (RhoA) and Ras-related C3 botulinum toxin substrate 1 (Rac1), suppress the dephosphorylation and degradation of synaptopodin, and directly ameliorate the injury to podocyte foot process barrier function ^64^. Based on this dual action, CNIs are commonly used in the management of glomerular diseases characterized by persistent proteinuria ^65^. Compared with CsA, TAC is associated with less hirsutism and coarsening of facial features. Results from recent clinical trials showed that dual or triple (termed “multitarget” in some studies) immunosuppressive regimens that included TAC or CsA were associated with comparable or higher short-term response rates, and often more rapid reduction of proteinuria, in the treatment of patients with active severe LN compared to control groups treated with standard immunosuppressive regimens that did not include a CNI ^66– 73^. With the level of proteinuria being a key component in the definition of treatment response, the findings are not unanticipated. One should be reminded that proteinuria is but a surrogate biomarker, one that is relatively easy to measure repeatedly, for long-term renal survival ^74^, and that the preservation of long-term kidney function is the ultimate objective in LN management ^75^. An alternative approach to inclusion of a CNI upfront in the treatment of active nephritis is to add the drug in patients whose proteinuria has not decreased to an acceptable low level after treatment with standard therapies, and our group has reported on the efficacy and safety of long-term TAC treatment adopting this approach ^76^. Both CsA and TAC are associated with marked individual variations in pharmacokinetics and the side-effects of nephrotoxicity and hypertension, among others, and discontinuation after a relatively short duration of treatment is associated with a high rate of relapse in proteinuric kidney diseases ^77, 78^. Questions that need to be answered regarding the role of TAC or CsA in the management of LN include the optimal level and duration of drug exposure, how to avoid nephrotoxicity, especially in patients with significant chronic kidney disease, and their impact on long-term clinical outcomes.

Voclosporin is a novel calcineurin inhibitor that is an analogue of CsA. Although voclosporin is structurally similar to CsA, a single carbon atom is added to the amino acid-1 region, resulting in enhanced binding to calcineurin and increased potency, faster elimination, and less plasma variation than CsA ^79– 81^. In a recent phase II clinical trial in active LN (AURA-LV; NCT02141672), voclosporin (high or low dose) when combined with corticosteroids and mycophenolate mofetil (MMF) showed superior renal response rates compared with corticosteroids and MMF induction at 24 weeks and 48 weeks ^82^. A subsequent phase III study (AURORA; NCT03021499) showed that voclosporin (23.7 mg bid) in combination with MMF and corticosteroids, the latter adopting a force-tapering reduced-dose regimen, achieved statistically superior and faster renal response rate (defined as a urinary protein-to-creatinine ratio [UPCR] of ≤0.5 mg/mg and an estimated glomerular filtration rate [eGFR] of ≥60 ml/minute or no decrease from baseline of >20%), compared with MMF and corticosteroid treatment ^83^. It is prudent to avoid over-immunosuppression in patients treated with triple immunosuppression, as suggested by the higher rate of infective complications in Chinese patients treated with the “multitarget” regimen ^67^ and the significant number of infection-related deaths in Asian patients in the AURA-LV trial ^82^.

## Emerging therapies targeting co-stimulatory pathways

Co-stimulation in T lymphocyte activation also plays an important role in autoimmunity. Co-stimulation requires accessory molecules such as CD28/CD80/CD86 and CD40/CD40L. The engagement of CD28 on the cell surface of naïve T cells by CD80 and CD86 present on antigen-presenting cells or B cells activates signaling pathways that promote clonal expansion, T cell survival, and release of pro-inflammatory cytokines and other mediators of inflammation ^84^. Once activated, T cells express cytotoxic T-lymphocyte-associated protein 4 (CTLA4), a co-stimulatory molecule homologous to CD28, which has a higher affinity to CD80 and CD86. CTLA4 serves as a negative regulator of T and B cell co-stimulation and terminates T cell response and cytokine secretion. CTLA4 serves as a master switch for peripheral T cell tolerance as demonstrated by massive lymphoproliferation and early death in CTLA4-knockout mice ^85^.

Abatacept (or CTLA4Ig) is a soluble fusion protein comprising the extracellular domain of CTLA4 and a modified fragment of the Fc domain of human IgG. It is a CTLA4 agonist that interrupts the interaction of CD80/CD86 with CD28 and thus inhibits T cell co-stimulation, thereby suppressing T cell activation and B cell response ^86^. Treatment of mice with CTLA4Ig reduced the expansion of IgG and IgM autoreactive B cell subsets and CD4 ^+^ T cells and suppressed Ig subclass switching, delayed disease onset and anti-dsDNA production, decreased the severity of proteinuria and renal damage, and prolonged survival ^84^. Combination treatment with CTLA4Ig and cyclophosphamide was more effective than CTLA4Ig alone in delaying the onset of nephritis in mice and was effective in reducing renal damage in mice with advance renal disease ^87^. A phase II clinical trial (NCT00430677) showed that induction therapy with abatacept combined with corticosteroids and MMF improved proteinuria and serological parameters in patients with active proliferative LN after 12 months but failed to meet the superiority efficacy endpoint (time to complete renal response) ^88^. Results from a phase III study (ALLURE; NCT01714817) showed that, in patients with active LN, treatment with abatacept on a background of corticosteroids and MMF for 2 years with a blinded long-term extension improved serological parameters with a more rapid improvement in proteinuria compared with controls who received corticosteroids and MMF with placebo, but the primary endpoint of demonstrating a higher complete remission rate based on clinical parameters in the abatacept group was not achieved ^89^. The negative results in both studies have been attributed to the relatively high efficacy of background induction treatment and perhaps over-stringent criteria for treatment response ^90^. It has been reported that the podocytes in patients and mice with proteinuric kidney diseases showed increased CD80 expression, which was associated with down-regulation of β1 integrin ^91, 92^. Therefore, apart from interrupting the interaction of CD80/CD86 with CD28, abatacept may stabilize podocyte cytoskeleton and reduce proteinuria through its effect on β1-integrin ^92^. Whether abatacept may be better suited to treat LN patients with increased CD80 expression in podocytes has not been investigated.

Blockade of the CD40/CD40L pathway presents an alternative therapeutic approach targeting co-stimulatory signals in T lymphocytes. A phase II clinical trial (NCT02770170) is underway to investigate the efficacy and safety of BI655064 (humanized anti-CD40 monoclonal antibody) in combination with standard-of-care therapy in active LN patients. Another phase II clinical trial (NCT03610516) will test the role of CFZ533 (a humanized anti-CD40 monoclonal antibody) in LN patients who show persistent significant proteinuria after standard induction treatments. Data from a recent phase II study (NCT02804763) demonstrated that dapirolizumab pegol (a pegylated Fab anti-CD40L) treatment in active SLE patients was associated with numerically higher response rates and greater improvement in several clinical outcome measures compared with placebo, but the difference in primary efficacy endpoint of British Isles Lupus Assessment Group-based Composite Lupus Assessment (BICLA) response between the two groups did not reach statistical significance ^93^.

## Emerging therapies targeting signaling pathways, pro-inflammatory cytokines, complement activation, or neutrophil extracellular traps

In SLE, multiple signaling pathways and inflammatory mediators are induced by cells of the innate and adaptive immune system and contribute to both systemic and local inflammation. Increases in type I IFN signature, which promotes B cell differentiation and loss of tolerance, and IL-17 and IL-23, which drive tissue injury, are prominent examples ^94^.

### Mammalian or mechanistic target of rapamycin

The mammalian or mechanistic target of rapamycin (mTOR) is an evolutionarily conserved serine-threonine kinase that plays a key role in the regulation of cell proliferation, metabolism, and survival. Increased mTOR activation is observed in patients and mice with LN ^95^. Sirolimus is a naturally occurring macrolide antibiotic produced by *Streptomyces hygroscopicus* and is a specific inhibitor of the mTOR pathway. It is a potent inhibitor of B and T cell proliferation. In pre-nephritic NZB/W F1 mice, treatment with sirolimus suppressed lymphoproliferation, reduced monocyte chemoattractant protein-1 (MCP-1) expression in the kidney, and delayed phenotypic manifestation of disease ^96^. Sirolimus treatment given after the onset of nephritis in NZB/W F1 mice was effective in decreasing anti-dsDNA level, improving kidney histopathology, and prolonging survival ^97– 99^. There is also emerging evidence that the anti-proliferative property of mTOR inhibitors extends beyond lymphocytes to non-immune cells. We recently demonstrated that sirolimus decreased mesangial cell proliferation and their binding by anti-dsDNA antibodies and suppressed fibrotic responses in mesangial cells when cells were exposed to anti-dsDNA antibodies or transforming growth factor (TGF)-β1, and the effect was mediated through down-regulation of mTOR and extracellular signal-regulated kinase phosphorylation ^95^. In NZB/W F1 mice with active nephritis, sirolimus was as effective as mycophenolate in attenuating kidney inflammation and fibrosis ^95^. A recent single-arm phase I/II study (NCT00779194) reported that sirolimus treatment in patients with active SLE but without nephritis or life-threatening lupus complications resulted in a decrease in disease activity score and a T cell profile indicating a less pro-inflammatory phenotype ^100^. We have also reported our experience of the use of sirolimus in LN patients, suggesting that this group of drugs may have a role in LN management, especially in patients who do not tolerate other treatments or who could derive benefit from the unique properties of these drugs, such as a lower rate of malignancies ^101, 102^.

### Janus kinase-1, tyrosine kinase-2, and Bruton’s tyrosine kinase signaling pathways

The success of Janus kinase (JAK) inhibition in murine lupus has prompted clinical trials on JAK inhibitors in human SLE. One phase II RCT (NCT02708095) demonstrated the efficacy of baricitinib (a JAK1/2 inhibitor) in alleviating arthritis in SLE patients ^103^. A phase IIb study (PF-06700841) will investigate a selective JAK1 inhibitor in moderate-to-severe active SLE patients without renal or cerebral involvement who show an inadequate response to standard therapies. Inhibition of JAK1 should be used with caution, as a phase II clinical trial on solcitinib (a selective JAK1 inhibitor) (NCT01777256) was discontinued because of severe drug reaction with eosinophilia and systemic symptoms (DRESS) syndrome and hepatic function abnormalities ^104, 105^. Tyrosine kinase 2 (TYK2) is a member of the JAK family and functions downstream of IL-12, IL-23, and type I IFN signaling. A phase II RCT (PAISLEY; NCT03252587) will evaluate the safety and efficacy of BMS-986165, a specific TYK2 inhibitor, in active SLE patients with joint and/or skin involvement ^106^, and patients who successfully complete the protocol-required treatment period will be recruited to a subsequent phase II trial that will investigate the long-term safety and efficacy (NCT03920267). Bruton’s tyrosine kinase (BTK) is a non-receptor tyrosine kinase expressed in B cells and myeloid cells, and it plays an important role in B cell development, survival, and activation and mediates TLR signaling in macrophages. Inhibition of BTK in lupus-prone mice after disease onset reduced total splenic B cell number and suppressed B cell activation, accompanied by decreased proteinuria and improvement of serological parameters ^107^. Treatment of moderate-to-severe active SLE patients without cerebral or renal involvement with the BTK inhibitor fenebrutinib reduced CD19 ^+^ B cells, anti-dsDNA antibody titer, and BTK-dependent signature in plasmablasts, but the primary clinical efficacy endpoint (SRI-4) was not met ^108^.

### Cytokines

The importance of type I IFN in SLE and LN is well established. Anifrolumab is a monoclonal antibody against type I IFN receptor subunit 1, and the results from a phase IIb clinical trial (MUSE) showed that anifrolumab treatment significantly suppressed type I IFN gene signatures and lupus disease activity ^109^. The results from two subsequent phase III RCTs (TULIP-1 and -2) also showed the clinical benefits of anifrolumab in moderate-to-severe active SLE patients. Although the primary endpoint (SRI-4) was not achieved in TULIP-1, anifrolumab treatment could facilitate corticosteroid reduction and improve disease activity scores such as the CLASI and BICLA scores ^110^. The results from TULIP-2 corroborated with TULIP-1: anifrolumab met its primary endpoint (BICLA scores at 52 weeks) in active SLE patients ^111^. Consistent with the scientific rationale, the clinical benefit of anifrolumab appears to be more evident in patients with high type I IFN signatures ^111^.

Given the pathogenic significance of the IL-17/IL-23 axis in SLE and LN, inhibition of these cytokines may potentially ameliorate SLE and LN. Secukinumab is a fully humanized IgG1κ monoclonal anti-IL-17A antibody and is an approved treatment for active psoriatic arthritis and ankylosing spondylitis, and a phase II clinical trial is underway to investigate its role in patients with refractory LN (NCT04181762). Guselkumab is an anti-IL-23 monoclonal antibody licensed for the treatment of plaque psoriasis, and its use in LN will be explored in a forthcoming phase II study. IL-2 regulates CD4 ^+^ T cell development and survival, and defective IL-2 production leads to dysregulation in the immune system in SLE patients ^112, 113^. Previous clinical trials demonstrated that treatment of SLE patients with low-dose IL-2 promoted regulatory T (Treg) cells and inhibited T _H_17 cells and was associated with the induction of remission ^114, 115^. One recent RCT showed that low-dose IL-2 treatment (1 million IU subcutaneously every other day for 2 weeks) in active SLE patients did not meet the primary efficacy endpoint of SRI-4 at 12 weeks but achieved higher remission rates than placebo in patients with LN and was well tolerated ^116^. The low-dose IL-2 group also showed expansion of Treg and natural killer cells ^116^.

### Complement cascade

The complement cascade plays an essential role in innate immunity and comprises more than 30 circulating proteins. The complement system can be activated through one of three initiation pathways, namely the classical pathway, mannose-binding lectin pathway, and the alternative pathway ^117^. The three pathways converge at C3, resulting in the cleavage of C3 to the activated components C3a and C3b. Dysregulation of complement activation is observed in patients and mice with LN. In LN, deposition of C3-containing immune complexes in the glomeruli initiates tissue injury, whereas deficiencies of some of the components of the classical complement pathway, such as C1q and C4, are associated with an increase in the incidence of lupus in humans and also lupus-like disease in C1q or C4 knockout mice ^118^. APL-2 (pegcetacoplan) is a small, synthetic cyclic peptide that binds and inhibits C3 and C3b and interferes with the formation of C3 and C5 convertases as well as inhibits the activity of pre-formed C3 and C5 convertases ^119^. A phase II clinical trial investigated the safety and efficacy of daily APL-2 subcutaneous infusion, administered for 16 weeks with a 6-month safety follow-up, in patients with various glomerulopathies including LN (DISCOVERY; NCT03453619). Preliminary experience from two LN patients recruited into this trial suggests that APL-2 is well-tolerated and treatment was associated with a reduction of proteinuria ^120^. Yet the pharmaceutical company has decided to stop the development program for APL-2 in LN after completion of the phase II trial and prefers to focus on other diseases ^121^.

Treatment of pre-nephritic NZB/W F1 mice with a monoclonal antibody that inhibits C5 cleavage to the potent pro-inflammatory and pro-thrombotic molecules C5a and C5b-9 delayed the development of proteinuria, decreased anti-dsDNA antibody level, improved kidney histopathology, and prolonged survival ^122^. Eculizumab is a recombinant fully humanized hybrid IgG2/IgG4 monoclonal antibody that directly binds human complement component C5 and prevents its cleavage to C5a and C5b-9, thereby blocking terminal complement activation while preserving the functions of early complement components. The success of inhibiting C5 cleavage in murine lupus has prompted a single-dose placebo-controlled phase I study to investigate the safety, pharmacodynamics, and pharmacokinetics of eculizumab in 24 SLE patients. While eculizumab at 4 or 8 mg/kg was well tolerated and complement level was reduced to baseline level 2 weeks after commencement of the study, clinical parameters were similar to the placebo group. Relatively low baseline disease activity was proposed as a possible reason for the lack of apparent clinical efficacy associated with eculizumab treatment ^123, 124^. A multicenter phase II clinical trial was designed to investigate the effect of eculizumab in patients with proliferative LN, but the study encountered logistic delays and was terminated ^125^. Eculizumab has been approved for the treatment of atypical hemolytic uremic syndrome (aHUS) to inhibit C5-mediated thrombotic microangiopathy ^126^. Eculizumab has been reported to be effective in the treatment of a patient with catastrophic antiphospholipid antibody syndrome ^127^ and also in a patient with refractory LN ^128^. It has been proposed that eculizumab may be beneficial in the treatment of LN patients who have a thrombotic component analogous to aHUS, evidence of C5-driven glomerular inflammation, or antiphospholipid syndrome, although further studies are warranted. It is noteworthy that inhibition of complement activation at the level of C5 is associated with *Neisseria* infection and prophylactic measures may be required ^118^.

Dysregulation of the classical complement pathway contributes to the pathogenesis of SLE, and there is emerging evidence to show that the alternative pathway also plays a significant role. The alternative pathway is triggered by the activation of factor B, a trypsin-like serine protease that is the proteolytically active component of C3 and C5 convertases. Factor B serves as an acute-phase protein and also as a B cell growth factor. Data from 222 Chinese patients with LN showed reduced plasma C1q and C3 levels and increased levels of factor B, C3a, C5a, and soluble C5b-9 ^129^. Plasma factor B level correlated with that of C5a and soluble C5b-9, and factor B colocalized with C3b and C5b-9 deposits in the glomeruli ^129^. MRL/lpr mice deficient in factor B showed reduced proteinuria, fewer active serological parameters, and improved kidney histology and vasculitis features compared to wild-type and heterozygous mice ^130^. The association between factor B deficiency and improved disease manifestation may be attributed to decreased C3 activation and/or B cell development. These data suggest that factor B may present a novel therapeutic target in LN ^130^. A highly potent, reversible, and selective small molecule factor B inhibitor (LNP023) is available ^131, 132^. Oral administration of LNP023 to mice with KRN-induced arthritis reduced overall clinical score and inhibited complement activation and infiltration of immune cells in the joints ^131^. In a rodent model of membranous nephropathy, LNP023 administration before disease onset suppressed the development of proteinuria, while treatment after disease onset delayed progression and attenuated glomerulopathy ^131^. Phase II clinical studies will investigate the safety, efficacy, tolerability, pharmacokinetics, and pharmacodynamics of LNP023 in patients with paroxysmal nocturnal hemoglobinuria (NCT03439839 and NCT03896152) and IgA nephropathy (NCT03373461).

OMS721 (narsoplimab) is a human monoclonal antibody that targets mannan-binding lectin serine protease 2 (MASP-2), the effector enzyme of the lectin pathway. A phase II study is ongoing to investigate the safety of OMS721 in patients with glomerular diseases including active LN and will look into its potential steroid-sparing effect (NCT02682407). Preliminary results appear encouraging, with treated patients showing a significant reduction of proteinuria (mean reduction 69%) and the feasibility for steroid tapering ^133^. A phase III study will investigate the effect of OMS721 in LN patients ^134^.

### Neutrophil extracellular traps

Neutrophils play a critical role in the innate immune response, and they also regulate the adaptive immune response ^135^. Neutrophil cell death, termed NETosis, results in the formation of NETs, which are composed of nuclear components such as DNA and histones decorated with proteins from primary, secondary, and tertiary granules such as myeloperoxidase, neutrophil elastase, pentraxin 3, and matrix metalloproteinase 9. Under physiological conditions, neutrophils release NETs as a defense mechanism to trap and kill microorganisms ^135^. NET formation can also be induced by sterile stimuli such as pro-inflammatory cytokines, immune complexes, or autoantibodies ^135, 136^. In SLE, NET formation is dysregulated and can be induced by ribonucleoprotein antibodies through FcγRIIB (also known as CD32) binding, by the antimicrobial peptide LL37, and by IFN-α produced by plasmacytoid dendritic cells ^137^. SLE-induced NETs are believed to be highly immunogenic and contain oxidized mitochondrial DNA, HMGB1, LL37, and immunostimulatory molecules ^137, 138^. The degradation and clearance of NETs in SLE patients are dependent on DNase 1, but DNase 1 activity is impaired in lupus because of the presence of either DNase 1 inhibitors or anti-NET antibodies that inhibit the access of DNase 1 to the NETs, and components of the increased NETs then trigger downstream tissue injury and inflammation ^135, 139, 140^. The inability to degrade NETs is associated with a higher propensity for LN ^140^. In lupus-prone mice, inhibition of NET formation using Cl-amidine (a chemical inhibitor of peptidylarginine deiminase-4) ^141^, DNase 1 ^142^, or FR139317 (a specific endothelin-A receptor antagonist) ^143^ resulted in delayed onset of disease, decreased proteinuria, and reduced deposition of immune complexes in the glomeruli as well as down-regulation of IFN-signature inflammatory responses in the bone marrow and kidney ^141– 143^, suggesting that targeting NETs may be beneficial to lupus patients. Tofacitinib, a JAK/STAT inhibitor, has been shown to reduce NET formation in lupus-prone mice, and this was associated with improvements in proteinuria and serological parameters ^144^. An ongoing phase I study is investigating the safety of tofacitinib in patients with SLE (NCT02535689). Combination therapy with rituximab and belimumab has also been shown to decrease immune complex-induced NET formation in SLE patients ^46^. Metformin has been shown to reduce NET formation in neutrophils stimulated with phorbol 12-myristate 13-acetate and decreased IFN-α secretion in plasmacytoid dendritic cells stimulated with mitochondrial DNA ^145^. Data from a study in Chinese patients (ChiCTR-TRC-12002419) showed that metformin added to conventional treatment for 12 months in SLE patients with mild-to-moderate disease resulted in a decrease in SLE disease activity index (SLEDAI) score by over 50% ^145^. Mitochondrial DNA is deposited in NETs in renal biopsy specimens from LN patients, and mitochondrial DNA has been shown to induce higher levels of IFN-α in plasmacytoid dendritic cells compared with anti-dsDNA antibodies. In the MUSE trial, SLE patients with a high type I IFN signature showed higher plasma levels of neutrophil granule constituents such as myeloperoxidase, human neutrophil elastase, and citrullinated histone H3 levels, and treatment with anifrolumab for 1 year resulted in a significant decrease in circulating neutrophil NET complexes compared to the placebo group ^146^.

## Conclusion and future direction

As a direct result of greater understanding of disease pathogenesis and key pathways and molecules causing immune activation or organ injury, the development and implementation of novel therapeutic agents for SLE and LN have grown substantially over the past decade. Yet, despite encouraging results from animal studies, relatively few novel therapies have successfully progressed to general clinical usage. Valuable lessons have been learnt from the clinical trials of these novel therapies. Indeed, many of these studies, including those which have apparently failed to achieve their intended aim of demonstrating superiority of the new drug compared with placebo, have provided clinical evidence that substantiate the scientific rationale supporting the clinical relevance of specific pathogenic pathways. Results from these trials, both the positive and the negative ones, have highlighted the heterogeneity in treatment response between different patient subgroups and the caveats and pitfalls in the design of clinical trials, resulting in improved trial design and therapeutic strategies. A challenge in the use of agents that target specific signaling pathways or cytokines is that some of the molecules could serve both pro-inflammatory and anti-inflammatory properties depending on the local cytokine milieu. Given that a multitude of effector mechanisms are activated during active disease, therapies that combine or target multiple pathways may be relevant, although the risk of over-immunosuppression could be a concern. Future trials should also explore ways of incorporating the new treatments to reduce the exposure to established but toxic therapies, especially corticosteroids.

## Abbreviations

aHUS, atypical hemolytic uremic syndrome; APRIL, a proliferating-inducing ligand; BAFF, B cell activating factor; BICLA, British Isles lupus assessment group-based composite lupus assessment; BTK, Bruton’s tyrosine kinase; CLASI, cutaneous lupus erythematosus disease area and severity index; CNI, calcineurin inhibitor; CRBN, cereblon; CsA, cyclosporine A; CTLA4; cytotoxic T-lymphocyte-associated protein 4; ds, double-stranded; IFN, interferon; Ig, immunoglobulin; JAK, Janus kinase; IL, interleukin; LOI, loss of improvement; LN, lupus nephritis; MMF, mycophenolate mofetil; mTOR, mammalian or mechanistic target of rapamycin; NETs, neutrophil extracellular traps; NFAT; nuclear factor of activated T cells; NZB/W F1, New Zealand black and white, first generation; PBMC, peripheral blood mononuclear cell; RCT, randomized controlled trial; SLE, systemic lupus erythematosus; SRI, systemic lupus erythematosus responder index; STAT, signal transducer and activator of transcription; TAC, tacrolimus; TACI, transmembrane activator and cyclophilin ligand interactor; TLR, Toll-like receptor; TNF, tumor necrosis factor; Treg, regulatory T; TYK2, tyrosine kinase-2.

## List of clinical studies and their acronyms

**Table:** 

ALLURE:	“Efficacy and Safety Study of Abatacept to Treat Lupus Nephritis” Trial [NCT00430677] ^89^
APRIL-LN:	“The Efficacy and Safety of Atacicept in Combination with Mycophenolate Mofetil Used to Treat Lupus Nephritis” Trial [NCT00573157] ^43^
AURA-LV:	“Aurinia Urinary Protein Reduction Active - Lupus with Voclosporin” Trial [NCT02141672] ^82^
AURORA:	“Aurinia Renal Response in Active Lupus with Voclosporin” Trial [NCT03021499] ^83^
BLISS-52:	“A Study of Belimumab in Subjects with Systemic Lupus Erythematosus (SLE)” Trial [NCT00424476] ^35^
BLISS-76:	“A Study of Belimumab in Subjects with Systemic Lupus Erythematosus (SLE)” Trial [NCT00410384] ^36^
BLISS-BELIEVE:	“A Study to Evaluate the Efficacy and Safety of Belimumab Administered in Combination with Rituximab to Adult Subjects with Systemic Lupus Erythematosus (SLE) - BLISS-BELIEVE” Trial [NCT03312907] ^47^
BLISS-LN:	“Efficacy and Safety of Belimumab in Patients with Active Lupus Nephritis (BLISS-LN)” Trial [NCT01639339] ^39^
CALIBRATE:	“Rituximab and Belimumab for Lupus Nephritis” Trial [NCT02260934] ^45^
ILLUMINATE-1:	“A Study of LY2127399 in Participants with Systemic Lupus Erythematosus” Trial [NCT01205438] ^40^
ILLUMINATE-2:	“A Study of LY2127399 in Participants with Systemic Lupus Erythematosus” Trial [NCT01196091] ^41^
LUNAR:	“A Study to Evaluate the Efficacy and Safety of Rituximab in Subjects with International Society of Nephrology/Renal Pathology Society (ISN/RPS) 2003 Class III or IV Lupus Nephritis” Trial [NCT00282347] ^22^
MISSION:	“A Study of KZR-616 in Patients with Systemic Lupus Erythematosus With and Without Nephritis” Trial [NCT03393013] ^51^
MUSE:	“A Study of the Efficacy and Safety of MEDI- 546 in Systemic Lupus Erythematosus” Trial [NCT01438489] ^109^
NOBILITY:	“A Study to Evaluate the Safety and Efficacy of Obinutuzumab Compared with Placebo in Participants with Lupus Nephritis (LN)” Trial [NCT02550652] ^33^
PAISLEY:	“An Investigational Study to Evaluate BMS- 986165 in Patients with Systemic Lupus Erythematosus” Trial [NCT03252587] ^106^
SynBioSe-1:	“Synergetic B-cell Immunomodulation in SLE” Trial [NCT02284984]46
SynBioSe-2:	“Synergetic B-cell Immunomodulation in SLE - 2 ^nd^ Study” Trial [NCT03747159]
TULIP-1:	“Efficacy and Safety of Anifrolumab Compared to Placebo in Adult Subjects with Active Systemic Lupus Erythematosus” Trial [NCT02446899] ^110, 111^
TULIP-2:	“Efficacy and Safety of Two Doses of Anifrolumab Compared to Placebo in Adult Subjects with Active Systemic Lupus Erythematosus” Trial [NCT02446912]
